# Calibrated interdental brushing for the prevention of periodontal pathogens infection in young adults - a randomized controlled clinical trial

**DOI:** 10.1038/s41598-019-51938-8

**Published:** 2019-10-22

**Authors:** Denis Bourgeois, Manuel Bravo, Juan-Carlos Llodra, Camille Inquimbert, Stéphane Viennot, Claude Dussart, Florence Carrouel

**Affiliations:** 1 Systemic Healthcare Laboratory EA4129, Faculty of Medicine Laennec, University Lyon 1, University of Lyon, Lyon, France; 2 Department of Preventive and Community Dentistry, Faculty of Oral Medicine, University of Granada, Granada, Spain; 3 Department of Public Health, Faculty of Oral Medicine, University of Montpellier, Montpellier, France; 4 Department of Fundamental and Clinical Biological Sciences, Faculty of Oral Medicine, University Lyon 1, University of Lyon, Lyon, France

**Keywords:** Oral hygiene, Dental biofilms

## Abstract

Periodontal disease is clearly correlated with systemic disease. The presence of periodontal pathogens in interdental spaces in young, healthy adults is a strong indicator of the need to introduce daily interdental prophylaxis. Twenty-five subjects (aged 18–35 years), diagnosticated clinically as periodontally healthy, were enrolled in this study. One hundred interdental sites were included. Among these sites, 50 “test” sites were cleaned daily with calibrated interdental brushes (IDBs), whereas the other 50 sites were not cleaned and considered “controls”. The interdental biofilm at these interdental sites was collected at the beginning of the study (basal) and at 1 week, 2 weeks, 3 weeks, 4 weeks, and 3 months. Real-time polymerase chain reaction (PCR) methodology was used to quantify (i) 19 periodontal bacteria, including *Porphyromonas gingivalis*, *Treponema denticola*, and *Tannerella forsythia*, and (ii) total bacteria. In the test sites, the quantity of total bacteria decreased over time with the use of IDBs. The bacteria from the red and orange Socransky complexes, which are associated with periodontal disease, significantly decreased in the test sites but not in the control sites. Bacteria from the yellow, and purple Socransky complexes, which are associated with periodontal health, increased significantly in both groups whereas bacteria from the blue Socransky complex increased significantly only in the test sites. Furthermore, at basal, 66% of test sites and 68% of control sites bled during interdental brushing. These percentages decreased by 85% in 3 months for the test sites and by 27% in the control sites. In conclusion, the daily use of calibrated IDBs can reduce periodontal pathogens, reestablish symbiotic microbiota and, decrease interdental inflammation in interdental sites of healthy young adults.

## Introduction

Periodontal disease (ICD-10 KO5.3), with a prevalence estimated at 750 847 000 cases in 195 countries and territories^1^, is an oral infectious disease caused by complex interactions between the microbial biofilm and host immune responses^2^. Periodontal pathogens can enter the host system through daily bacteremia following breakdown of epithelial and endothelial barriers due to the host’s inflammatory responses and the ability of some pathogens to attack these barriers^3^. However, the importance of oral-systemic connection is not only due to the implication of periodontal pathogens in the pathogenesis of periodontitis and systemic diseases. The presence of periodontal pathogens and their metabolic by-products in the mouth may modulate the immune response beyond the oral cavity, thus promoting the development of systemic inflammation and thus systemic disease^4^.

Periodontal disease exhibits an association with multiple systemic diseases, such as cardiovascular disease (including atherosclerosis, stroke, hypertension, myocardial infarction, and congestive heart failure), rheumatoid arthritis, inflammatory bowel disease and colorectal cancer, respiratory tract infection, type 2 diabetes mellitus, and respiratory tract infection^5–7^. From a clinical point of view, it is evident that the prevention of periodontal disease strategies directed at keystone pathogens could have a major effect on the incidence and progression of systemic disease^8^.

Periodontal pathogens were categorized into color-coded complexes based on their role in periodontal pathogenesis^9^. Red complex bacteria (*Porphyromonas gingivalis*, *Treponema denticola*, *Tannerella forsythia*) are the major etiologic agents that contribute to a high risk of chronic periodontitis by modulating the host inflammatory response^10^. Orange complex bacteria, including *Fusobacterium nucleatum*, *Prevotella intermedia*, *Prevotella nigrescens*, *Parvimonas micra*, *Eubacterium nodatum*, and various *Campylobacter* species, are classified as moderate risk^11^. Blue (*Actinomyces viscosus*, etc.), yellow (*Streptococcus mitis*, *Streptococcus spp*., etc.), green (*Aggregatibacter actinomycetemcomitans*, *Campylobacter concisus*, *Capnocytophaga ochracea*, *Capnocytophaga sputigena*, *Eikenella corrodens*, etc.), and purple (*Actinomyces odontolyticus*, *Veillonella parvula*, etc.) complexes are compatible with periodontal health^9^.

In 2016, pathogens were identified in interdental spaces in young adults with healthy periodontium. *P*. *gingivalis* was detected in 19%, *T*. *denticola* in 49%, and *T*. *forsythia* in 93% of healthy young adults, whereas pathogens from the orange complex were detected in at least 81% of these subjects. The major explanation is that interdental spaces are a unique and real ecological niche, for which the body has few or no alternative defenses and where traditional daily hygiene methods are not adequate for disrupting biofilm^12^.

This effective presence of virulent pathogens in healthy young adults is a strong indicator of the need to initiate new methods for disrupting interdental biofilm in daily oral hygiene. Recently, the study of Duval and colleagues has suggested that the “everyday low-level bacteremia” that occurs after toothbrushing, flossing, interdental brushing or chewing could also potentially pose risk to endocarditis^13^. Therefore, the use of interdental brushes (IDBs) could represent a serious alternative to reduce the level of virulent bacteria and to optimize the disruption of biofilms. To our knowledge, no randomized controlled clinical trial has shown the microbiological impact of calibrated IDBs on the interdental virulent microbiota in healthy adults. The aim of the present study was therefore to determine the bacterial efficacy of the use of calibrated IDBs on red and orange complex bacteria in young adults without chronic periodontal complaints.

## Methods

### Study design

**Figure 1:**
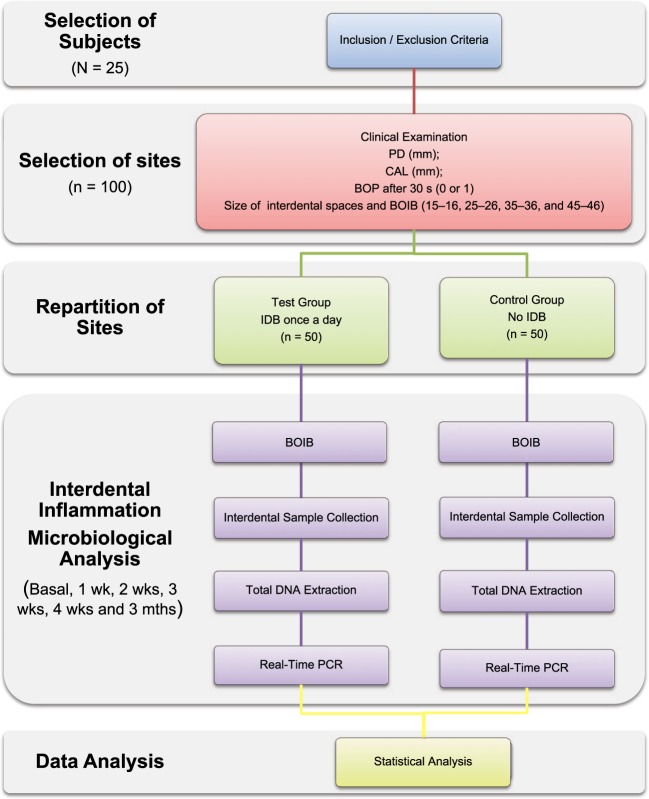
Workflow of the experiment. BOIB: Bleeding on Interdental Brushing; BOP: Bleeding on Probing; CAL: Clinical Attachment Loss; IDB: Interdental Brush; PD: Probing Depth.

The workflow of the impact of interdental brushing on the evolution of interdental biofilm (BACTERIB) is described in Fig. 1.

### Study population

This study included 25 Caucasian adults aged 20 to 35 years old. Subjects were recruited from a pool of first-time volunteers who were referred to the Department of Public Health of the Faculty of Oral Medicine at the University of Lyon, France. All participants signed an informed consent form in accordance with the Declaration of Helsinki. The study protocol was reviewed and approved by the Local Ethics Committee “University Hospital Center of Lyon” (Rech_FRCH_2015-0181) and by the National Commission of Informatics and Liberties, France (1845681v0). This study was retrospectively registered with ClinicalTrials.gov (identification number: NCT03714295).

Subjects were included if they were aged between 20 and 35 years, in good general health, not pregnant or breastfeeding, and periodontally healthy and if they had no health conditions that required antibiotic prophylaxis before interproximal probing, no experience with interdental cleaning (interdental brushing or dental flossing), and no intake of systemic antimicrobials during the previous 6 months. To be included, the subjects had to brush their teeth at least twice per day, not use chlorhexidine or over-the-counter mouthwash, have no implants or orthodontic appliances, have no previous periodontal illness or treatment history, have at least 24 natural teeth, have 4 premolar-molar pairs, be non-smokers, and have a willingness to return 3 weeks after the clinical investigation for microbiological tests.

The clinical inclusion criteria for each premolar-molar interdental site were as follows: (i) accessibility to the interdental space of the 4 sites (15–16, 25–26, 35–36, and 45–46) by an interdental brush in each subject; (ii) no interproximal caries or dental or prosthetic restorations; (iii) no interdental diastema; (iv) no clinical signs of inflammation, such as redness, swelling; (v) no pocket depth (PD) > 3 mm or clinical attachment loss (CAL) > 3 mm; and (iv) the subjects were judged to be free of gingivitis or periodontitis.

Subjects were excluded if they had missing teeth due to periodontal issues, any other concomitant systemic disorder, diseases affecting the immune system, and use of medications, such as anti-platelet or anti-coagulant agents. The patients submitted to professional prophylaxis 4 weeks prior to the baseline examination; patients with previous periodontal disease or treatment or who were undergoing a course of dental or orthodontic treatment were also excluded.

### Classification of subjects as periodontally healthy

The diagnosis of periodontally healthy was made according to the American Academy of Periodontology^14^, with some modifications^15^. The patients were scored for PD, CAL, and BOP. The studied group was composed of individuals who presented with clinically healthy periodontal tissues (PD ≤ 3 mm, CAL < 3 mm and ≤10% of sites with BOP after 30 s). Clinical assessments of 4 interdental sites (15–16, 25–26, 35–36, and 45–46) were performed using an IAP CURAPROX colorimetric probe (Curaden, Kriens, Switzerland) (Supplementary Fig. 1). The diameter and bleeding upon interdental brushing (BOIB) were recorded^16^.

### Clinical examination

Standardized clinical monitoring was performed three weeks before the microbiological monitoring. The subjects were submitted to a medical/dental anamnesis, and information regarding their age, gender and smoking status was obtained. The clinical measurements were performed at 6 sites per tooth (mesio-buccal, buccal, disto-buccal, disto-lingual, lingual, and mesio-lingual) on all teeth, except for the third molars, as previously described^17^. The clinical parameters were measured in the following order: PD (mm), CAL (mm), and BOP after 30 s (0 or 1). The full-mouth clinical measurements included BOP, PD and CAL, which were recorded using a North Carolina periodontal probe (Hu-Friedy, Chicago, IL, USA).

The clinical assessments of the interdental spaces were performed using an IAP CURAPROX^©^ colorimetric probe (Curaden, Kriens, Switzerland). The diameter of all interdental spaces of 4 teeth (premolar-molar) was measured to determine the calibrated IDB (diameter of the IDB corresponding to the interdental space diameter). Subjects received IDBs (Curaprox CPS; Curaden) of sizes corresponding to the diameter of their interdental spaces. The first use of the product was conducted under the supervision of a qualified public health professor. The instruction comprised verbal instructions on interdental brushing supported by practical demonstration. No further oral-hygiene instructions were provided. All other brushings were unsupervised, and the participants were required to maintain a diary card. Subjects were instructed to mark the box corresponding to the current date on the diary card every evening after performing their interdental brushing to ensure that brushing was performed every day. Moreover, the participants were instructed to brush their teeth 3 hours before the sampling visit and not to drink, eat or practice oral hygiene during this period.

### Interdental site randomization

For each patient, 4 interdental sites localized between premolar-molar (15–16, 25–26, 35–36, and 45–46) were studied. Among these 4 sites studied, 2 were designed as test sites and 2 were designed as control sites. The test sites (2 per patient, total n = 50) and the control sites (2 per patient, total n = 50) were randomized symmetrical on each upper and lower arch (Table 1). One test site and one control site were in the upper jaw and even at the level of the mandible. Patients were instructed to brush their teeth twice a day for 2 min, rinse with water. In the test sites after brushing their teeth, patients were trained to use calibrated interdental brushes for a single cleaning movement once a day (in the evening) only. No other product or oral hygiene technique was allowed.

**Table 1:** Repartition of Sites (n = 100^a^) According to the Quadrant.

Quadrant	Control sites	Test sites	Total of sites
1	12	13	25
2	13	12	25
3	13	12	25
4	12	13	25
Total of sites	50	50	100

^a^25 patients with 1 site by quadrant (4 sites by patients).

### Interdental sample collection and microbiological analysis by real-time polymerase chain reaction

At each clinical examination, the same four interdental sites (15–16, 25–26, 35–36, and 45–46) were studied (total of 100 sites) in all subjects. Based on the clinical assessment of the interdental spaces, the examiner selected the appropriate CPS prime interdental brushes (Curaden, Kriens, Switzerland)^18^. He isolated each previously selected tooth with sterile cotton rolls, and removed the interdental biofilm with a sterile, calibrated IDB. For each sample, he placed the IDBs in 1.5 mL sterile microcentrifuge tubes and stored at 4 °C for further processing. Then, the examiner recorded the BOIB^16^. For the test sites, the collection was performed at the following six time points: at baseline, 1, 2, 3, and 4 weeks and 3 months; for the control sites, the collection was not performed at 2, 3, and 4 weeks to avoid perturbations due to the introduction of the IDB for the sampling.

The total DNA was extracted using QIAcube HT Plasticware and a Cador Pathogen 96 QIAcube HT Kit (Qiagen, Hilden, Germany) according to manufacturer’s guidelines. The DNA was eluted in 150 µL. DNA quantities and quality were determined with an ultraviolet spectrophotometer at 260 nm and 280 nm.

Real-time polymerase chain reaction (PCR) was used to detect and quantify the total bacterial load (TB) and 19 periodontal pathogens (red complex: *P*. *gingivalis*, *T*. *forsythia*, *T*. *denticola;* orange complex: *C*. *gracilis*, *C*. *rectus*, *F*. *nucleatum*, *P*. *intermedia*, *P*. *micra*, *P*. *nigrescens;* yellow complex: *S*. *mitis*, *Streptococcus spp*.*;* green complex: *E*. *corrodens*, *C*. *sputigena*, *C*. *ochracea*, *C*. *concisus*, *A*. *actinomycetemcomitans;* purple complex: *V*. *parvula*, *A*. *odontolyticus*; and blue complex: *A*. *viscosus*) in interdental biofilms. Real-time PCR was performed as previously described by Carrouel and colleagues^12^. The sequences of universal primers for the 16S rRNA genes and species-specific primer sets used are shown in the Supplementary Table 1.

For each sample, simplex quantitative PCR (qPCR) was performed in a total volume of 10 µL using qPCR kit (1 × SYBR^®^ Premix Ex Taq^TM^ Tli RNaseH Plus, TaKaRa, Shiga, Japan), which contained 2 μL of the template DNA and 1 µM of each primer. A Rotor-Gene^®^ Q thermal cycling system (Qiagen, Hilden, Germany) was used to performed the assays. The cycle for each sample was the following program: 95 °C for 30 s, followed by 40 cycles of 10 s at 95 °C, 10 s at the annealing temperature (Supplementary Table 1), and 35 s at 72 °C. A final melting curve analysis (70 °C to 95 °C in 1 °C steps at 5 s increments) was performed. For every cycle, at the end of the extension step and continuously during the melting curve analysis, the fluorescence signal was measured. The Rotor-Gene^®^ Q Series software (Qiagen, Hilden, Germany) was used to analyze the data.

Serial dilutions of a bacterial standard DNA provided by Institut Clinident SAS (Aix en Provence, France) were used in each reaction as external standards for the absolute quantification of the targeted bacterial pathogens. The standard bacterial strains came from BCMM/LMG Bacteria Collection, CIP Collection of Institut Pasteur, or from DSMZ (Germany): *Aa* (DSM No. 8324), *Ao* (DSM No. 43760), *Av* (DSM No. 43327), *Cc* (DSM No. 9716), *Cg* (DSM No. 19528), *Co* (DSM No. 7271), *Cr* (LMG No. 7613), *Cs* (DSM No. 7273), *Ec* (DSM No. 8340), *Fn* (DSM No. 20482), *Pg* (DSM No. 20709), *Pi* (DSM No. 20706), *Pm* (DSM No. 20468), *Pn* (DSM No. 13386), *Smitis* (DSM No. 12643), *Sspp* (*Streptococcus mitis* DSM No. 12643), *Td* (DSM No. 14222), *Tf* (CIP No. 105220), and *Vp* (CIP No. 60.1). The Supplementary Table 1 details the limit of quantification (LOQ).

### Statistics

The statistical analysis consisted of three main steps, namely, producing descriptive summaries of the data, modeling the data using a mixed (linear) model and assessing the correlations between the bacterial abundances. Descriptive statistics (percentages, means and standard deviations) were calculated with SPSS 12.0 (SPSS Inc., Chicago, IL). The statistical tests (p-values) were calculated with SUDAAN 7.0 (Research Triangle Institute, Research Triangle Park, NC) to account for clustering (multiple sites within the mouth) and repeated measures (measurements along the follow-up period). Statistical methods are clearly indicated in table footnotes. All data were considered statistically significant at p < 0.05.

## Results

### Periodontal parameters in the patients

The study sample was composed of 10 females and 15 males (Table 2). The clinical parameters confirmed that subjects were periodontally healthy. A comparison of the test sites with the control sites revealed similar scores for BOP, PD, CAL and ID diameter (p > 0.05).

**Table 2:** Baseline Clinical Features of the Subjects, Test Sites, and Control Sites.

Subjects			Means comparison (p-value)^a^
Full mouth			
Sampled sites	Test Sites	Control Sites	
**Age (years)**	26.8 ± 4.6		
**Sex**, **n** (**%**)			
Male	15 (60.0)		
Female	10 (40.0)		
**Teeth**	28.9 ± 1.2		
BOP (%)	0.16 ± 0.08		
PD (mm)	0.95 ± 0.21		
CAL (mm)	0.95 ± 0.21		
BOP (%)	0.10 ± 0.02	0.10 ± 0.01	≈1
PD (mm)	1.37 ± 0.28	1.32 ± 0.23	≈1
CAL (mm)	1.60 ± 0.41	1.58 ± 0.40	≈1
**ID diameter**			
0.6 mm, n (%)	1 (2.0)	2 (4.0)	
0.7 mm, n (%)	12 (24.0)	28 (56.0)	
0.8 mm, n (%)	21 (42.0)	12 (24.0)	
0.9 mm, n (%)	12 (24.0)	5 (10.0)	
1.1 mm, n (%)	4 (8.0)	3 (6.0)	
mean ± sd (mm)	0.76 ± 0.12	0.76 ± 0.11	≈1

The values are means ± standard deviations.

BOP: Bleeding On Probing; CAL: Clinical Attachment Level; ID: Interdental; PD: Pocket Depth. ^a^By SUDAAN 7.0.

### Evolution of interdental bleeding

**Figure 2:**
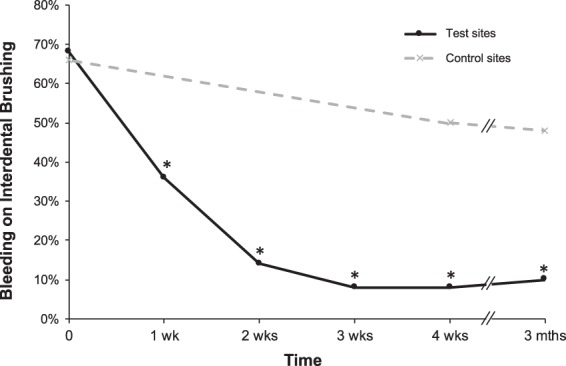
Evolution of interdental bleeding over time. The results are expressed as the percentage of the 50 test (daily use of calibrated interdental brushes) or 50 control (no use of interdental brushes) sites presenting bleeding on interdental brushing. *p-value < 0.05 for pairwise comparison vs T0.

The bleeding during interdental brushing decreased by 47% after one week of the daily use of IDBs and 85% after 3 months in the test sites, whereas no significant change was observed in the control sites (Fig. 2 and Table 3).

**Table 3:** Evolution of Interdental Bleeding in the Test and Control Sites.

	Basal (T0)	4 weeks (T4)	3 months (T5)
Test sites	68%	8%	10%
Control sites	66%	50%	48%

The results are expressed as the percentage of active or control sites that bled compared to the total of active or control sites.

### Quantitative analysis of socransky’s complexes

Table 4 shows that bacteria from the green, orange and red Socransky complexes were significantly decreased over time in subjects using IDBs, in contrast to subjects not using IDBs. Bacteria from the blue complex significantly increased in the test sites but not in the control sites, whereas bacteria from the purple, and yellow complexes increased in both type of sites. The main differences between groups up to 3 months are on orange and red complexes.

**Table 4:** Evolution of Bacteria in the Socransky Complexes in the Test and Control Sites.

Bacteria	Test sites N = 50			Control sites N = 50		
	Basal (T0)	4 weeks (T4)	3 months (T5)	Basal (T0)	4 weeks (T4)	3 months (T5)
**Total bacteria**	10.08 ± 0.38	9.85 ± 0.42*	9.77 ± 0.53*	10.00 ± 0.39	10.05 ± 0.46	10.02 ± 0.51
**Socransky’s Blue** (*Actinomyces viscosus*)	0.77 ± 1.41	0.82 ± 1.28	1.35 ± 1.85*	0.63 ± 1.29	0.42 ± 0.97	1.24 ± 1.77
**Socransky’s Purple**	5.75 ± 0.69	6.50 ± 0.52*	6.75 ± 0.63*	5.67 ± 0.70	6.32 ± 0.62*	6.68 ± 0.59*
*Veillonella parvula*	5.66 ± 0.74	6.45 ± 0.55*	6.74 ± 0.63*	5.55 ± 0.77	6.29 ± 0.64*	6.66 ± 0.58*
*Actinomyces odontolyticus*	4.27 ± 1.23	4.95 ± 0.68*	4.33 ± 1.86*	4.10 ± 1.47	4.52 ± 1.14	4.40 ± 1.49
**Socransky’s Green**	6.80 ± 0.75	6.52 ± 0.99*	6.25 ± 0.83*	6.74 ± 0.80	6.61 ± 0.91	6.82 ± 0.94
*Eikenella corrodens*	6.44 ± 0.98	6.08 ± 1.39*	5.73 ± 1.22*	6.20 ± 1.38	5.82 ± 1.38*	5.46 ± 1.56*
*Capnocytophaga sputigena*	5.03 ± 1.05	5.17 ± 1.90	4.86 ± 1.90	4.89 ± 1.23	4.63 ± 2.02	4.68 ± 1.75
*Capnocytophaga ochracea*	5.78 ± 1.30	4.45 ± 2.40*	4.72 ± 2.04*	5.66 ± 1.45	5.34 ± 2.03	5.21 ± 1.82
*Campylobacter concisus*	3.23 ± 1.54	3.46 ± 1.80	3.51 ± 1.31*	3.16 ± 1.47	3.58 ± 1.62*	3.79 ± 1.34*
*Aggregatibacter actinomycetemcomitans*	0.24 ± 1.21	0.35 ± 1.44	0.28 ± 1.14	0.50 ± 1.70	0.61 ± 1.90	0.53 ± 1.65
**Socransky’s Yellow**	6.29 ± 0.57	6.58 ± 0.47*	6.61 ± 0.75*	6.16 ± 0.58	6.46 ± 0.53*	6.46 ± 0.66*
*Streptococcus mitis*	5.09 ± 0.75	5.10 ± 0.77	5.22 ± 0.73	4.94 ± 0.73	4.81 ± 0.85	5.02 ± 0.77
*Streptococcus spp*.	6.25 ± 0.56	6.55 ± 0.47*	6.58 ± 0.76*	6.13 ± 0.57	6.44 ± 0.53*	6.43 ± 0.67*
**Socransky’s Orange**	7.80 ± 0.48	7.16 ± 0.76*	7.05 ± 0.87*	7.71 ± 0.48	7.73 ± 0.54	7.61 ± 0.63
*Campylobacter gracilis*	4.79 ± 1.08	3.77 ± 1.76*	3.72 ± 1.70*	4.77 ± 0.97	4.88 ± 0.73	4.51 ± 1.33
*Campylobacter rectus*	6.41 ± 1.89	5.77 ± 1.68*	5.88 ± 1.18*	6.54 ± 1.37	6.91 ± 2.06	6.77 ± 2.18
*Prevotella intermedia*	5.63 ± 2.76	3.88 ± 2.86*	4.18 ± 2.73*	5.47 ± 2.87	4.78 ± 2.89	5.06 ± 2.72
*Prevotella nigrescens*	3.94 ± 1.62	1.16 ± 1.80*	1.07 ± 1.81*	3.92 ± 1.78	3.66 ± 1.98	3.02 ± 2.02
*Parvimonas micra*	5.30 ± 1.98	4.41 ± 2.50*	4.99 ± 2.04	4.94 ± 2.21	4.79 ± 2.21	5.06 ± 2.17
*Fusobacterium nucleatum*	7.57 ± 0.36	7.01 ± 0.77*	6.85 ± 0.95*	7.45 ± 0.35	7.51 ± 0.50	7.47 ± 0.61
**Socransky’s Red**	6.02 ± 2.11	3.63 ± 2.62*	4.13 ± 2.45*	5.89 ± 1.86	5.54 ± 2.07	5.29 ± 2.42
*Porphyromonas gingivalis*	1.17 ± 2.50	0.89 ± 1.01*	0.76 ± 0.62*	1.11 ± 2.47	1.04 ± 2.14	0.99 ± 2.06
*Tannerella forsythia*	5.75 ± 1.93	3.43 ± 2.39*	3.58 ± 2.47*	5.66 ± 1.72	5.31 ± 1.88	5.22 ± 2.36
*Treponema denticola*	3.47 ± 3.47	1.78 ± 2.82*	2.59 ± 3.02*	3.02 ± 3.38	2.79 ± 3.40	2.50 ± 3.33

N: Number of interdental sites; *p-value < 0.05 for pairwise comparison vs T0 by SUDAAN 7.0 (procedure DESCRIPT) to account for clustering (multiple sites within the patient).

The results are expressed as the mean ± standard deviation after Log conversion (Log_10_ (count + 1)) of bacterial counts.

**Figure 3:**
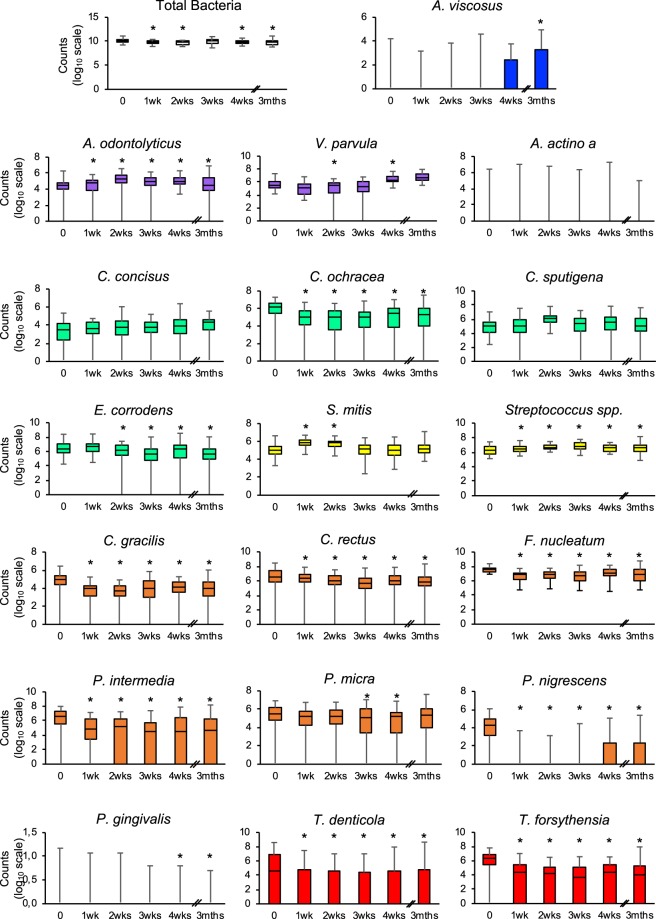
Evolution of the abundance of bacterial species over time in the test sites. The counts are reported on a log_10_ scale. Each box represents the first quartile, median quartile, and third quartile, from bottom to top. The box colors represent the colors of the Socransky complexes. *p-value < 0.05 for pairwise comparison vs T0 by SUDAAN 7.0 (procedure DESCRIPT) to account for clustering (multiple sites within the patient).

Figure 3 and Table 5 show that the daily use of IDBs makes it possible to significantly reduce the number of orange and red complex bacteria in as little as a week, except for *P*. *micra* and *P*. *gingivalis*. The quantity of *P*. *micra* is significantly reduced after 3 weeks of daily IDB use, while for *P*. *gingivalis*, it takes 4 weeks.

**Table 5:** Evolution of Bacteria in the Socransky Complexes in the Test Sites.

Bacteria^a^	Time						Comparison^b^	
	Basal (T0)	1 week (T1)	2 weeks (T2)	3 weeks (T3)	4 weeks (T4)	3 months (T5)	Global p-value	Pairwise *vs* T0 p<0.05
**Total bacteria**	10.08 ± 0.38	9.77 ± 0.33	9.69 ± 0.34	9.94 ± 0.47	9.85 ± 0.42	9.77 ± 0.53	<0.001	T1, T2, T4, T5
**Socransky's Blue** (*Actinomyces viscosus*)	0.77 ± 1.41	0.41 ± 0.95	0.43 ± 1.02	0.58 ± 1.20	0.82 ± 1.28	1.35 ± 1.85	<0.001	T5
**Socransky's Purple**	5.75 ± 0.69	5.36 ± 0.75	5.73 ± 0.56	5.54 ± 1.10	6.50 ± 0.52	6.75 ± 0.63	<0.001	T1, T4, T5
*Veillonella parvula*	5.66 ± 0.74	5.08 ± 0.95	5.01 ± 1.20	5.10 ± 1.42	6.45 ± 0.55	6.74 ± 0.63	<0.001	T1, T2, T3, T4, T5
*Actinomyces odontolyticus*	4.27 ± 1.23	4.27 ± 1.43	5.08 ± 1.25	4.72 ± 1.54	4.95 ± 0.68	4.33 ± 1.86	<0.001	T2, T4
**Socransky's Green**	6.80 ± 0.75	6.66 ± 0.81	6.60 ± 0.72	6.19 ± 0.85	6.52 ± 0.99	6.25 ± 0.83	<0.001	T3, T4, T5
*Eikenella corrodens*	6.44 ± 0.98	6.51 ± 0.90	6.06 ± 1.19	5.25 ± 1.82	6.08 ± 1.39	5.73 ± 1.22	<0.001	T2, T3, T4, T5
*Capnocytophaga sputigena*	5.03 ± 1.05	5.84 ± 1.67	5.02 ± 0.92	5.12 ± 1.84	5.17 ± 1.90	4.86 ± 1.90	0.068	—
*Capnocytophaga ochracea*	5.78 ± 1.30	4.59 ± 1.80	4.30 ± 2.16	4.28 ± 2.27	4.45 ± 2.40	4.72 ± 2.04	<0.001	T1, T2, T3, T4, T5
*Campylobacter concisus*	3.23 ± 1.54	3.28 ± 1.44	3.53 ± 1.54	3.43 ± 1.51	3.46 ± 1.80	3.51 ± 1.31	0.045	—
*Aggregatibacter actinomycetemcomitans*	0.24 ± 1.21	0.36 ± 1.47	0.39 ± 1.38	0.33 ± 1.34	0.35 ± 1.44	0.28 ± 1.14	0.365	—
**Socransky's Yellow**	6.29 ± 0.57	6.65 ± 0.49	6.75 ± 0.36	6.90 ± 0.52	6.58 ± 0.47	6.61 ± 0.75	<0.001	T1, T2, T3, T4, T5
*Streptococcus mitis*	5.09 ± 0.75	5.87 ± 0.45	5.71 ± 0.46	5.07 ± 0.88	5.10 ± 0.77	5.22 ± 0.73	<0.001	T1, T2
*Streptococcus spp*.	6.25 ± 0.56	6.55 ± 0.52	6.69 ± 0.38	6.89 ± 0.52	6.55 ± 0.47	6.58 ± 0.76	<0.001	T1, T2, T3, T4, T5
**Socransky's Orange**	7.80 ± 0.48	7.05 ± 0.62	7.03 ± 0.65	6.82 ± 0.78	7.16 ± 0.76	7.05 ± 0.87	<0.001	T1, T2, T3, T4, T5
*Campylobacter gracilis*	4.79 ± 1.08	3.67 ± 1.30	3.42 ± 1.44	3.67 ± 1.85	3.77 ± 1.76	3.72 ± 1.70	<0.001	T1, T2, T3, T4, T5
*Campylobacter rectus*	6.41 ± 1.89	6.07 ± 1.72	5.61 ± 2.02	5.24 ± 1.93	5.77 ± 1.68	5.88 ± 1.18	<0.001	T1, T2, T3, T4, T5
*Prevotella intermedia*	5.63 ± 2.76	4.23 ± 2.60	4.20 ± 2.80	3.34 ± 3.00	3.88 ± 2.86	4.18 ± 2.73	0.002	T1, T2, T3, T4, T5
*Prevotella nigrescens*	3.94 ± 1.62	0.66 ± 1.28	0.53 ± 1.09	0.80 ± 1.56	1.16 ± 1.80	1.07 ± 1.81	<0.001	T1, T2, T3, T4, T5
*Parvimonas micra*	5.30 ± 1.98	5.10 ± 1.61	4.96 ± 1.60	4.40 ± 2.28	4.41 ± 2.50	4.99 ± 2.04	0.017	T3, T4
*Fusobacterium nucleatum*	7.57 ± 0.36	6.70 ± 0.62	6.82 ± 0.65	6.66 ± 0.80	7.01 ± 0.77	6.85 ± 0.95	<0.001	T1, T2, T3, T4, T5
**Socransky's Red**	6.02 ± 2.11	3.91 ± 2.55	3.72 ± 2.53	3.21 ± 2.74	3.63 ± 2.62	4.13 ± 2.45	<0.001	T1, T2, T3, T4, T5
*Porphyromonas gingivalis*	1.17 ± 2.50	1.08 ± 2.28	1.08 ± 2.24	0.83 ± 2.00	0.89 ± 1.01	0.76 ± 0.62	0.242	T4, T5
*Tannerella forsythia*	5.75 ± 1.93	3.57 ± 2.37	3.40 ± 2.36	3.00 ± 2.50	3.43 ± 2.39	3.58 ± 2.47	<0.001	T1, T2, T3, T4, T5
*Treponema denticola*	3.47 ± 3.47	1.89 ± 2.83	2.02 ± 2.78	1.95 ± 2.81	1.78 ± 2.82	2.59 ± 3.02	<0.001	T1, T2, T3, T4, T5

^a^Bacteria (mean ± standard deviation) after LOG conversion (Log10 (count + 1)) of bacteria counts.

^b^By SUDAAN 7.0 (procedure REGRESS for global p-value and DESCRIPT for pairwise comparison only if global p-value is < 0.05) to account for clustering (multiple sites within the patient).

Table 6 confirms that the daily use of IDBs impacts the number of bacteria from the orange and red complexes at 4 weeks and 3 months, whereas no significant differences were observed at baseline.

**Table 6:** Comparison of 50 Control and 50 Test Interproximal Sites after Follow-up in 25 Patients.

Bacteria	Time		
	Basal (T0)	4 weeks (T4)	3 months (T5)
**Total bacteria**	0.286	0.004	0.273
**Socransky's Blue** (*Actinomyces viscosus*)	0.453	0.002	0.662
**Socransky's Purple**	0.269	0.027	0.339
*Veillonella parvula*	0.138	0.040	0.282
*Actinomyces odontolyticus*	0.360	0.036	0.851
**Socransky's Green**	0.590	0.956	0.968
*Eikenella corrodens*	0.170	0.284	0.207
*Capnocytophaga sputigena*	0.413	0.049	0.435
*Capnocytophaga ochracea*	0.516	0.022	0.169
*Campylobacter concisus*	0.652	0.725	0.602
*Aggregatibacter actinomycetemcomitans*	0.158	0.095	0.118
**Socransky’s Yellow**	0.083	0.131	0.220
*Streptococcus mitis*	0.068	0.002	0.100
*Streptococcus spp*.	0.104	0.172	0.242
**Socransky’s Orange**	0.296	<0.001	<0.001
*Campylobacter gracilis*	0.901	<0.001	0.054
*Campylobacter rectus*	0.550	0.683	0.680
*Prevotella intermedia*	0.741	0.108	0.132
*Prevotella nigrescens*	0.927	<0.001	0.008
*Parvimonas micra*	0.051	0.101	0.787
*Fusobacterium nucleatum*	0.096	<0.001	<0.001
**Socransky’s Red**	0.531	<0.001	<0.001
*Porphyromonas gingivalis*	0.819	0.043	0.036
*Tannerella forsythia*	0.592	<0.001	0.002
*Treponema denticola*	0.240	0.021	0.818

The results are expressed as p-values obtained by SUDAAN 7.0 (procedure DESCRIPT) to account for clustering (multiple sites within the patient).

The results from the multiple regression models (Table 7) indicate that the use of IDBs, the quantity of bacteria at T0, the age and the IDB diameter at 3 months have a significant statistical effect on the quantity of some bacteria. Sex had no effect.

**Table 7:** Analysis of Factors Impacting the Modification of Bacteria.

Bacteria	Test β ± se	Basal value β ± se	Age (yrs) β ± se	Male β ± se	IDB diam. (mm.) at 3 months β ± se
**Total bacteria**	−0.41 ± 0.07*	0.46 ± 0.16*	0.05 ± 0.01*	0.11 ± 0.11	0.05 ± 0.36
**Socransky’s Blue** (*Actinomyces viscosus*)	0.02 ± 0.26	0.64 ± 0.14*	−0.05 ± 0.04	0.28 ± 0.36	−0.22 ± 1.19
**Socransky’s Purple**	0.04 ± 0.08	0.35 ± 0.13*	0.02 ± 0.02	−0.10 ± 0.20	0.22 ± 0.59
*Veillonella parvula*	0.05 ± 0.08	0.30 ± 0.12*	0.02 ± 0.02	-0.14 ± 0.20	0.32 ± 0.63
*Actinomyces odontolyticus*	−0.16 ± 0.36	0.46 ± 0.10*	0.04 ± 0.04	0.05 ± 0.32	0.93 ± 1.33
**Socransky’s Green**	−0.05 ± 0.12	0.51 ± 0.13*	0.06 ± 0.02*	−0.00 ± 0.17	0.85 ± 0.71
*Eikenella corrodens*	0.11 ± 0.18	0.59 ± 0.15*	0.05 ± 0.03	0.01 ± 0.26	0.66 ± 1.04
*Capnocytophaga sputigena*	0.08 ± 0.22	0.40 ± 0.26	0.09 ± 0.05	−0.17 ± 0.46	3.08 ± 2.53
*Capnocytophaga ochracea*	−0.57 ± 0.30	0.76 ± 0.16*	0.01 ± 0.05	−0.38 ± 0.48	−0.91 ± 1.55
*Campylobacter concisus*	0.10 ± 0.22	0.29 ± 0.11*	0.05 ± 0.03	−0.17 ± 0.31	−0.68 ± 1.04
*Aggregatibacter actinomycetemcomitans*	−0.05 ± 0.17	0.77 ± 0.07*	0.03 ± 0.03	0.21 ± 0.17	−0.05 ± 0.55
**Socransky’s Yellow**	0.09 ± 0.11	0.39 ± 0.14*	0.03 ± 0.02	0.03 ± 0.19	0.68 ± 0.69
*Streptococcus mitis*	0.11 ± 0.10	0.38 ± 0.13*	0.03 ± 0.02	−0.02 ± 0.20	1.25 ± 0.80
*Streptococcus spp*.	0.09 ± 0.11	0.39 ± 0.14*	0.04 ± 0.02	0.04 ± 0.19	0.66 ± 0.72
**Socransky’s Orange**	−0.60 ± 0.13*	0.63 ± 0.10*	0.06 ± 0.01*	−0.09 ± 0.13	−1.40 ± 0.40*
*Campylobacter gracilis*	−0.58 ± 0.31	0.43 ± 0.19*	0.05 ± 0.03	−0.41 ± 0.31	−0.94 ± 1.38
*Campylobacter rectus*	0.17 ± 0.32	0.55 ± 0.13*	0.02 ± 0.03	−0.23 ± 0.40	1.01 ± 1.14
*Prevotella intermedia*	−0.90 ± 0.48	0.40 ± 0.10*	0.13 ± 0.05*	0.04 ± 0.47	−2.54 ± 1.57
*Prevotella nigrescens*	−0.97 ± 0.33*	0.46 ± 0.08*	0.16 ± 0.04*	0.15 ± 0.36	0.48 ± 1.01
*Parvimonas micra*	-0.29 ± 0.26	0.63 ± 0.10*	0.10 ± 0.03*	0.60 ± 0.38	-0.24 ± 1.11
*Fusobacterium nucleatum*	−0.65 ± 0.14*	0.52 ± 0.20*	0.07 ± 0.02*	−0.12 ± 0.17	−1.37 ± 0.52*
**Socransky’s Red**	−1.00 ± 0.27*	0.77 ± 0.13*	0.13 ± 0.05*	0.19 ± 0.41	−3.00 ± 1.14*
*Porphyromonas gingivalis*	−0.92 ± 0.13*	0.58 ± 0.08*	0.01 ± 0.02	0.03 ± 0.24	−1.78 ± 0.91
*Tannerella forsythia*	−1.10 ± 0.35*	0.70 ± 0.14*	0.14 ± 0.05*	0.32 ± 0.45	−1.60 ± 1.43
*Treponema denticola*	−0.71 ± 0.24*	0.54 ± 0.08*	0.14 ± 0.07*	−0.14 ± 0.62	−9.11 ± 2.19*

Multiple regression models^a^ (one model per bacterial count^b^ at the end of follow-up −3 months- as the dependent variable, -first column-). Data from 100 interproximal sites from 25 patients. Predictor variables (first line) are forced into the models.

^a^By SUDAAN 7.0 (procedure REGRESS) to account for clustering (multiple sites within the patient).

^b^Bacteria after LOG conversion (Log10 (count +1)) of bacteria counts.

*p < 0.05.

## Discussion

There is currently, no clinical research trial evaluating interdental prophylaxis options in clinically healthy young adults in relation to the presence of highly virulent periodontal bacteria. Effectively, only two studies compared toothbrushing plus interdental brushing to toothbrushing alone and reported data at one month. Both were at risk of bias^19^. Outcomes measured gingivitis by gingival index and by proportion of bleeding sites^20,21^. Moreover, Graziani and colleagues measured periodontitis but no data were reported^20^. So, prophylactic options are empirical and mainly arise from the experiences obtained during mechanical or surgical treatment of clinical periodontitis^22^. Our study is a Randomised Controlled Trials that compared a home-use interdental cleaning device and toothbrushing versus toothbrushing alone (duration 3 months) with an outcome variable, the quantitative evolution of periodontal bacteria. The results indicate that the daily use of calibrated IDBs, especially compared to simple toothbrushing, modifies the composition of interdental microbiota that becomes symbiotic and reduces interdental inflammation.

Periodontitis are multifactorial oral disease mainly due to bacteria and more particularly to the dysbiosis of the oral microbiota^23^. The symbiotic host-microbe relationship gradually changing to a pathogenic one. The periodontal health deteriorates until a state of clinical disease occurs. Simultaneously, a succession of microbial complexes develops. The biofilm associated with healthy mouth, mainly localised to enamel surfaces and oral mucosa, is composed of gram-positives aerobic bacteria such as bacteria from the blue, yellow, green, and purple complexes^24^. As undisturbed biofilm matures, the biofilm population changes to a predominately gram-negative anaerobic microbiota. Bacteria from the orange complex progressively adhere followed by bacteria from the red complex which are considered as “keystone pathogens”^25^. These periodontal pathogens are able to exploits complement-TLR crosstalk to subvert host defences and escape elimination. This defective immune monitoring results in a change in the biofilm composition (dysbiosis) that causes inflammatory periodontitis^26^.

Effective oral hygiene is crucial for maintaining good oral health, which is associated with global health^5^. Inside the oral sphere, the interdental gingiva, which is composed of the facial and lingual papillae and the col, is a unique area anatomically and histologically. The gingival stratified epithelia act as a barrier that serves to protect the internal tissues from environmental stresses, chemical damage, bacterial infection and antimicrobial protection. Moreover, the gingival col in the interdental space is not enhanced by keratinization^27,28^. In addition to the direct tissue destructive properties, some subgingival bacteria have different abilities to inhibit and interact with cells and components of the immune system to interfere with host reactions. Indeed, periodontopathogenic bacteria produce virulent factors that directly (enzymes and toxins) or indirectly (antigens and activators) induce an inflammatory response^29–31^. The virulence of bacteria depends on adhesion’s factor such as adhesins, lectins, fimbriae and vesicles. Moreover, periodontal bacteria produce agents that damage the periodontal tissues such as proteases, alkali and acid phosphatases, fatty and organic acids, igG- and igA-proteases, chondroitinsulfatase and toxic products (endotoxins, leukotoxin, mucopeptides of the bacterial wall, end-products of metabolism such as H_2_S, NH_4_, indole). All anatomical and physiological conditions are gathered for dissemination in the body of virulent bacteria by breaking the microcirculatory bloodstream^32^.

This study demonstrated that the interdental biofilm in young adults with no signs of gingivitis or periodontitis have all the characteristics favoring the chronic infection low-grade process. Inflammation has classically been viewed as an acute response to tissue injury that produces characteristic symptoms and usually resolves spontaneously. If the development of periodontal disease is a result of the concerted action of the total biofilm community, there is no biologically perfect state of health called “pristine gingiva”. Like the concept of para-inflammation developed in cancerology (Aran) i.e., a low-grade process identified with a gene signature, a para-inflammatory mechanism may be associated with periodontitis^33–35^. These proinflammatory instigators promote a perpetual low-level chronic inflammatory state^36^. Although it progresses silently, para-inflammation presents a major threat to the health and longevity of all aging humans.

Thus, by targeting the myriad of physiological variables that can elicit an inflammatory response, one can effectively prevent chronic inflammation and reduce the risk of inflammatory diseases. It is advantageous to keep bacterial reservoirs, such as those in the mouth, at the lowest levels possible to reduce the chances of the development of infection and chronic diseases. Regardless of age and medical history, reduction in infectious risks by oral virulent bacteria should be a priority.

In this study, young adults with no signs of gingivitis or periodontitis housed bacteria from the Socransky complexes and more particularly from the red and orange complexes in their interdental spaces. This result is consistent with what we observed in our previous study^12^. The presence of these highly virulent periodontopathogens is evidence of interdental dysbiotic microbiota. Although complex interactions between immune response mediators and biofilms are necessary for disease progression from gingivitis to periodontitis^37^, the host response is modified by dysbiotic microbiota, which provokes an inappropriate and uncontrolled level of inflammation^5^. This local inflammation causes an increased flow of nutrient-rich gingival crevicular fluid and potentially bleeding. Consequently, the inflamed site is deprived of oxygen, favoring the growth of anaerobic bacterial periodontopathogens^38^.

Moreover, in this study, healthy young adults with no clinical signs of gingivitis bleed when they use calibrated IDBs as we previously described^12^. They have inflammation in the interdental spaces, indicating that periodontal pathogens can penetrate the bloodstream^39^. Generally, oral bacteria are eliminated from the vascular system within 30 min and do not cause health problems^40^. However, in some cases, these microorganisms impact distant sites provoking disease. Even if periodontal pathogens promote development of non-oral disease directly or indirectly, migration of oral pathogens, related to the accumulation of biofilm, to the blood stream could also occur, causing higher risk to certain conditions^4^. Bacteremia has in fact been observed following some dental or medical procedures, and some bacteria were isolated from the blood after endodontic treatment. No relationship between the state of oral health and the incidence of bacteraemia due to dental extraction, toothbrushing and chewing was found^41^. The fact that the gingival tissue of the interdental space papilla neck is not keratinized reinforces the hypothesis of a metastatic spread of infection in the absence of gingivitis or periodontal disease. The particular histology of this area could also promote metastatic inflammation from the effects of circulating oral microbial toxins, and metastatic inflammation caused by immunological injury induced by oral microorganisms^32^. Thus, effective oral hygiene is a crucial factor in maintaining good oral health, which is associated with global health^5^.

Unfortunately, toothbrushing is ineffective in removing interdental biofilm even when the teeth are in a normal position^42^. Dental floss has been recommended for many years in conjunction with toothbrushing for removing dental plaque between teeth. IDBs have been developed for the same use, and many people find them easier to use than floss, providing there is sufficient space between the teeth^43^. However, to date, there is insufficient clinical evidence to determine whether interdental brushing reduces or increases the levels of plaque compared to flossing and/or toothbrushing^19^. Moreover, all these studies focused on patients who have gingivitis or periodontitis. Consequently, interdental prophylaxis in clinically healthy subjects remains to be improved.

Individual oral clinical prophylaxis is actually based on mechanical disruption and not on elimination of the biofilm^5^. To disrupt interdental biofilms, the entire interdental space must be in contact with the IDB filaments^12^. The key factor in efficient interdental prophylaxis is the choice of an IDB whose diameter must correspond to that of the interdental space. In our study, a colorimetric interdental probe was used to choose the calibrated IDBs^44^. The clinical use of a calibrated colorimetric probe for the measurement of interdental space diameters definitely adds value for decision-calibrated IDB support and could permit better action for the disruption of interdental biofilms^44^.

Our study demonstrates that the use of IDBs in addition to toothbrushing decrease significantly the total number of bacteria at 4 weeks and 3 months and, re-establish the symbiotic microbiota by decreasing the quantity of bacteria associated with periodontal health (the purple, yellow and blue complexes) and decreasing the quantity of bacteria associated with periodontal disease (the orange and red complexes). The toothbrushing alone permit to stabilize the quantity of total bacteria and the quantity of bacteria from the yellow and the red complexes at 4 weeks and 3 months. So, the toothbrushing alone allows over a period of 3 months to maintain a microbiota whose composition and compatible with a periodontal health. This confirms that periodontal dysbiosis occurs over a broadened timeframe, which slowly turns the symbiotic association of host and microbe to pathogenic^44^.

Our results indicate that the quantity of total bacteria decreases within one week of use of IDBs. In particular, bacteria from the red and orange complexes i.e., virulent pathogens, are significantly decreased after one week of IDB use; the only exception was *P*. *gingivalis*, which significantly decreased after 4 weeks. This delay in the response time could be explained by the fact that this major agent in the incidence of periodontal disease can be resistant^45^. First, *P*. *gingivalis* can escape to the host defense mechanisms, particularly when it is organized into a biofilm^46^. In fact, *P*. *gingivalis* can deregulate the host immune system by producing a number of virulence factors, such as lipopolysaccharide, fimbriae, and several proteases^47^. Interestingly, *T*. *denticola* is known to produce succinate, which facilitates the growth of *P*. *gingivalis*^48^, and could be the first bacterium eradicated. Second, *P*. *gingivalis* can be difficult to eradicate even with the use of antimicrobials^49^. Ardila and colleagues demonstrated that *P*. *gingivalis* strains isolated from patients suffering from periodontitis can be resistant to penicillin, clindamycin, amoxicillin, metronidazole, tetracycline, and azithromycin^50^. If antimicrobials are inefficient, periodontitis becomes chronic, and surgical treatment becomes necessary^46^. The daily use of IDBs could be a simple means to help fight against *P*. *gingivalis* and the other periodontal bacteria from the orange and red complexes.

Many studies have reported a link between bacteria from the red and orange complexes and systemic diseases^50^. For example, *P*. *gingivalis*, *T*. *forsythia*, *F*. *nucleatum*, and *T*. *denticola* can cause arteriosclerotic vascular diseases^6^. *P*. *gingivalis* in the respiratory mucosa increases the risk of developing aspiration pneumonia^51^. *P*. *gingivalis*, *T*. *forsythia* and *P*. *intermedia* are associated with poor glycemic control in diabetes mellitus patients^52^. Therefore, the daily use of calibrated IDBs, which leads to a decrease in the quantity of periodontal pathogens and interdental inflammation, could also be efficient in preventing some systemic diseases.

Concerning bacteria from the green complex, no significant effect was observed for *C*. *sputigena* and *A*. *actinomycetemcomitans*, whereas *E*. *corrodens*, *C*. *ochracea* and *C*. *concisus* were significantly decreased. *E*. *corrodens* was classified in a complex associated with microbiota symbiosis. *E*. *corrodens* might play an important role not only in healthy patients but also in the occurrence or progression of periodontitis in young patients^53^. *C*. *ochracea* and *C*. *concisus* are described in both healthy and diseased patients, underlying the possibility for strains to have different pathogenic potential^54,55^. Therefore, the pathogenic potential may differ depending on the anatomical site in the same host^56^. Depending on the ecological niche, bacteria adapt, leading to varying phenotypic expression and, consequently, different host responses^57^.

Bacteria from the blue, purple and yellow complexes, which are associated with oral health, increase over time with the daily use of IDBs. Our results demonstrate that, with the daily use of IDBs, the proportion of bacteria associated with good oral health increased, whereas bacteria associated with periodontal disease decreased. Moreover, these results are associated with a decrease in interdental inflammation. Therefore, the use of calibrated-diameter IDBs appears to be a key factor in disturbing the interdental biofilm and restoring the symbiosis of the microbiota.

Our study has several limitations. First, a targeted group of virulent bacteria has been identified mainly because of historical evidence of their ability to differentiate periodontitis from health. A subsequent prospective longitudinal experimental design in conjunction with a broader panel of bacteria could be necessary to demonstrate the prognostic ability of IDBs and link bacteria to non-oral diseases. Therefore, some of these results can only be considered exploratory, and a direct connection between local and systemic immunological factors cannot be assumed. A more detailed study that explicitly accommodates the various factors that can contribute to patient-specific variations may be necessary to assess the individual contributions of these factors. Further studies should follow-up on the immunological and microbiological results to characterize the connection between systemic factors and local factors in the periodontium more precisely.

Despite these limitations, this study is unique. We conducted a cohort study involving clinically healthy patients performed by professional dentists using the same criteria to minimize the errors introduced by clinical diagnosis and bacterial samples. One of the main strengths of this study is that we included well-defined cohorts with a number of sites adapted to the use of the PCR method and the incidence of virulent bacteria in healthy adults. Second, our study investigated a large number of sites to reflect a close-to-real-life-picture of the utilization of IDBs. The potential confirmation that natural gingival inflammatory processes are extravascular stimuli triggering systemic inflammation could have great public health implications.

In this context, our clinical results should introduce new clinical and public health perspectives.

In the short term, the daily use of IDBs could lead to the reestablishment of symbiotic interdental microbiota and the disappearance of interdental inflammation by decreasing periodontal pathogens. This could also contribute to reestablishing symbiosis of the salivary and oral microbiota.

In the medium term, this method could significantly reduce the incidence and/or severity of periodontal diseases. Toothbrushing is the cornerstone of dental health education to prevent periodontal disease, while also emphasizing the need to systematically clean all interdental tooth surfaces^58^. In fact, 90% of adults in industrialized countries report brushing twice a day^59^. However, the incidence of severe periodontitis in 2016 was 89 840 000 cases^1^. This prophylactic approach may be important in preventing and controlling chronic periodontitis by preventing growth and colonization by periodontal pathogens and decreasing inflammation.

In the long term, the daily use of calibrated IDBs should contribute to reducing risk, morbidity and mortality mainly related to systemic diseases such as cardiovascular diseases, diabetes, cancers and chronic respiratory diseases. To improve this hypothesis, long-term prospective cohort studies are needed.

## Conclusions

In this article, we performed a microbiological evaluation of a prophylactic technique used to reduce the incidence of periodontitis and systemic disease. This field of research is especially relevant considering the increasing morbidity and relatively high mortality previously discussed. Our study contributes to the production of information regarding strategies and research investigating the reduction of inflammation characteristic to specific health conditions.

Therefore, benefits in terms of oral prophylactic behaviors and general health should be observed considering the positive impact of using calibrated IDBs.

Effective collaboration that supports prophylactic activity must include participation by the whole community, including health professionals, dentists, patients and the wider community, and be focused on achieving results. In summary, calibrated interdental brushes are a great alternative to flossing that dental professionals can offer to patients to improve their health.

### Ethics approval and consent to participate

The study protocol was reviewed and approved by the Local Ethics Committee “University Hospital Center of Lyon” (Rech_FRCH_2015-0181) and by the National Commission of Informatics and Liberties, France (1845681 v 0). All participants signed a consent form in accordance with the Declaration of Helsinki. This study was registered with ClinicalTrials.gov (identification number: NCT03714295).

## Supplementary information

Calibrated interdental brushing for the prevention of periodontal pathogens infection in young adults - a randomized controlled clinical trial

## Data Availability

The authors thank all study subjects for their participation and the clinicians for their contributions leading to the successful completion of this study.
