# Supplementary material for: Calibrated interdental brushing for the prevention of periodontal pathogens infection in young adults - a randomized controlled clinical trial
Source: Sci Rep. 2019 Oct 22;9:15127. doi: 10.1038/s41598-019-51938-8 (PMC6805917; doi:10.1038/s41598-019-51938-8)

Calibrated interdental brushing for the prevention of periodontal pathogens infection in young adults - a randomized controlled clinical trial

Denis Bourgeois, Manuel Bravo, Juan-Carlos Llodra, Camille Inquimbert, Stéphane Viennot, Claude Dussart, Florence Carrouel

Supplementary Table 1. Species-specific and ubiquitous real-time PCR primers for 19 periodontal bacteria, the annealing temperature, and the limit of quantification.
LOQ: Limit of quantification; TB: Total Bacterial count.

Target	Primer pairs (5'-3')	References	Annealing temp (°C)	LOQ (E+02)
TB	CCATGAAGTCGGAATCGCTAGT<br>GCTTGACGGGCGTGTG	Kozarov et al., 2006	66	200
Aa	AAACCCATCTCTGAGTTCTTCTTC<br>ATGCCAACTTGACGTTAAAT	Kobayashi et al., 2008	60	10
Ao	CTTTGGGATAACGCCGGGAAAC<br>CTACCCGTCAAAGCCTTGGT	Yang et al., 2007	66	5
Av	ATGTGGGTCTGACCTGCTGC<br>CAAAGTCGATCACGCTCCG	Suzuki et al., 2005	60	5
Cc	GGCTCAAAAGAGATCGCTCA<br>CCCTCAACAACGCTTAGCTC	Chaban et al., 2009	66	5
Cg	AGAGTTTGATCCTGGCTCAG<br>GGACGCATGCCCATCTTTCACCACCGC	Kobayashi et al., 2008	66	5
Co	AGAGTTTGATCCTGGCTCAG<br>GATGCCGCTCCTATATACTATGGGG	Kobayashi et al., 2008	66	5
Cr	TTTCGGAGCGTAAACTCCTTTTC<br>TTTCTGCAAGCAGACACTCTT	Kobayashi et al., 2008	60	20
Cs	AGAGTTTGATCCTGGCTCAG<br>GATGCCGCTCCTATATACCATTAGG	Kobayashi et al., 2008	66	5
Ec	GGGAAGAAAAGGGAAGTGCT<br>TCTTCAGGTACCGTCAGCAAAA	Kozarov et al., 2006	60	50
Fn	AGAGTTTGATCCTGGCTCAG<br>GTCATCGTGCACACAGAATTGCTG	Fouad et al., 2002	60	40
Pi	CGTGGACCAAAGATTCATCGGTGG<br>ACCGCTTTACTCCCCAACAAA	Fouad et al., 2002	60	60
Pm	AGAGTTTGATCCTGGCTCAG<br>ATATCATGCGATTCTGTGGTCTC	Fouad et al., 2002	60	60
Pn	ATGAAACAAAGGTTTTCCGGTAAG<br>CCCACGTCTCTGTGGGCTGCGA	Fouad et al., 2002	66	5
Smitis	GAGTCCTGCATCAGCCAAGAG<br>GGATCCACCTTTTCTGCTTGAC	Suzuki et al., 2005	66	5
Sspp	AGAGTTTGATCCTGGCTCAG<br>GTACCGTCACAGTATGAACTTTCC	Fouad et al., 2002	66	10
Td	TAATACCGAATGTGCTCATTTACAT<br>TCAAAGAAGCATTCCCTCTTCTTCTTA	Sakamoto et al., 2001	60	10
Tf	GCGTATGTAACCTGCCCGCA<br>TGCTTCAGTGTCAGTTATACCT	Sakamoto et al., 2001	60	80
Vp	GAAGCATTGGAAGCGAAAGTTTCG<br>GTGTAACAAGG-GAGTACGGACC	Igarashi et al., 2009	60	5

Supplementary Figure 1.
(A) Colorimetric probe, (B) Interdental brushes, (C) Interdental space examination, (D) Interdental brushing with a calibrated interdental brush.

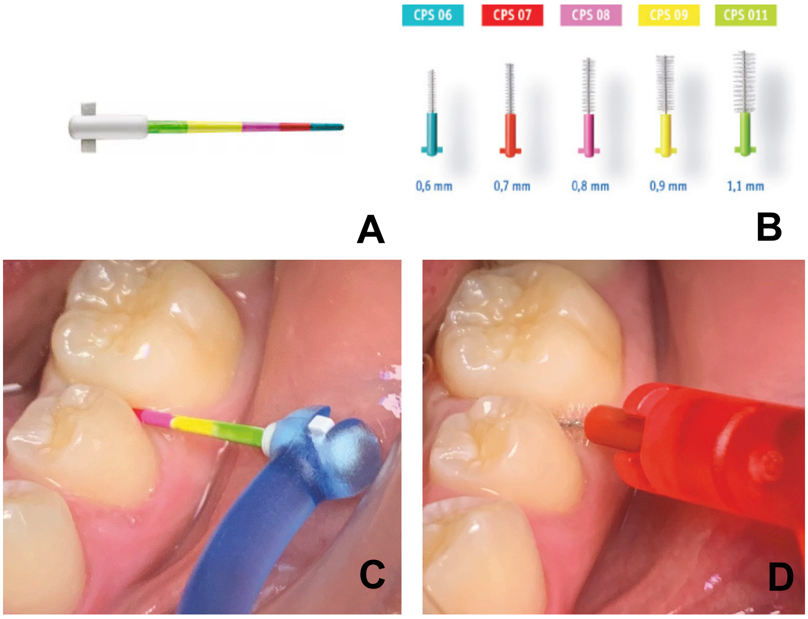

Supplement: Supplementary file 1 — Calibrated interdental brushing for the prevention of periodontal pathogens infection in young adults - a randomized controlled clinical trial [file 41598_2019_51938_MOESM1_ESM.pdf]
